# Peripheral immune and kynurenine profiles are associated with cognitive change during early treatment of first-episode schizophrenia

**DOI:** 10.3389/fpsyt.2026.1791018

**Published:** 2026-03-25

**Authors:** Jin-lei Tang, Fan-Fan Xu, Lian Wu

**Affiliations:** 1 Department of Psychiatry, Wenzhou Seventh People’ s Hospital, Wenzhou, Zhejiang, China; 2 The Second Affiliated Hospital, Yuying Children’s Hospital of Wenzhou Medical University, Wenzhou, Zhejiang, China

**Keywords:** cognition, first-episode schizophrenia, inflammation, kynurenine pathway, monocytes

## Abstract

**Objective:**

To delineate the longitudinal relationships among peripheral inflammation, kynurenine pathway (KP) dysregulation, immune-cell redistribution, and cognitive performance in antipsychotic-naïve first-episode schizophrenia (FES).

**Methods:**

Antipsychotic-naïve FES patients (n = 136) and healthy controls (HC; n = 136) were enrolled. FES received aripiprazole monotherapy through week 6, followed by naturalistic treatment through month 6. Cognition was assessed with the MATRICS Consensus Cognitive Battery (MCCB). Plasma KP metabolites were quantified by LC–MS/MS, inflammatory markers by high-sensitivity ELISA, and immune-cell subsets by flow cytometry.

**Results:**

FES showed marked baseline cognitive impairment with stepwise improvement by week 6 and month 6. At baseline, FES demonstrated a KP shift toward a relatively more neurotoxic profile (lower TRP and KYNA, higher KYN/TRP, 3-HK, and QA, with elevated QA/KYNA and reduced KYNA/3-HK), heightened inflammation (higher hs-CRP and pro-inflammatory cytokines with lower IL-10), and monocyte-skewed redistribution (higher CD14^+^ and CD16^+^ monocyte measures). Over follow-up, KP and inflammatory markers partially normalized and immune-cell distributions shifted toward the HC baseline profile. In FDR-adjusted longitudinal models, higher between-person inflammation and monocyte burden were associated with lower MCCB scores, whereas IL-10 and a more protective KP profile (higher KYNA, KYNA/KYN, and KYNA/3-HK) were associated with better cognition. In change-score analyses, ΔMCCB tracked KP remodeling—positively with ΔKYNA and ΔKYNA/KYN at both intervals and with ΔKYNA/3-HK at month 6, and inversely with Δ3-HK.

**Conclusions:**

Antipsychotic-naïve FES shows marked cognitive impairment with heightened inflammation, monocyte shifts, and KP dysregulation. Over follow-up, cognitive performance was more closely aligned with stable between-person immuno-KP differences, while cognitive improvement most consistently tracked KP remodeling rather than broad inflammatory or cellular change.

## Introduction

Schizophrenia is a debilitating psychiatric disorder with a complex and heterogeneous pathophysiology (1). It affects ~24 million people worldwide, with a global age-standardized prevalence and incidence of 287.4 and 16.31 per 100,000 persons, respectively (2, 3). Clinically, it comprises positive and negative symptoms alongside prominent cognitive dysfunction (4). Among these dimensions, cognitive dysfunction has become a major focus of contemporary schizophrenia research, given its strong association with daily functioning and quality of life (5, 6). Patients with first-episode schizophrenia (FES) commonly demonstrate broad neurocognitive deficits, including slowed processing speed, impaired attention/vigilance, disrupted working memory, learning deficits, and weaknesses in executive functions and social cognition, with processing speed and attention often among the most affected domains (7, 8). Therefore, identifying peripheral biomarkers at illness onset and mapping their early longitudinal dynamics may improve mechanistic understanding and inform future prognostic or treatment stratification efforts (9, 10).

A growing body of evidence implicates immune dysregulation in schizophrenia, particularly during early illness (11). Low-grade systemic inflammation and altered cytokine signaling have been repeatedly described, and peripheral immune activity may influence brain function through neuroimmune pathways such as microglial activation, modulation of blood–brain barrier integrity, and downstream effects on neurotransmission and synaptic plasticity (12, 13). In addition to soluble inflammatory mediators, immune perturbations can be reflected in shifts of circulating immune-cell subsets, including monocyte-related redistribution suggestive of innate immune engagement (14). Given that cognition is sensitive to inflammatory and innate immune activity in other neuropsychiatric contexts, peripheral cytokine profiles and immune-cell phenotypes may represent biologically plausible correlates of cognitive heterogeneity in FES.

The kynurenine pathway (KP) provides a mechanistic bridge linking inflammation to neurocognitive function via tryptophan metabolism. Tryptophan is predominantly catabolized through the KP, generating multiple neuroactive metabolites with immunomodulatory properties (15). The initial, rate-limiting step is catalyzed by indoleamine 2,3-dioxygenase (IDO), an enzyme inducible by pro-inflammatory cytokines including IFN-γ, IL-6, IL-1β, and TNF-α. Downstream metabolism yields compounds with divergent biological effects: kynurenic acid (KYNA) is often considered relatively neuroprotective, whereas 3-hydroxykynurenine (3-HK) and quinolinic acid (QA/QUIN) have been linked to oxidative stress and excitotoxic processes (16). KP dysregulation and altered balances between these metabolites have been proposed to contribute to neuropsychiatric and neurodegenerative disorders, including schizophrenia (4, 17). Beyond serving as inflammatory correlates, kynurenines are increasingly viewed as functional mediators of behavior and cognition; preclinical studies suggest that KP activation can contribute to behavioral phenotypes relevant to depression and schizophrenia-like cognitive deficits (18, 19). Despite this rationale, key gaps remain. Prior clinical studies have often been cross-sectional, have focused primarily on symptom severity rather than cognition, or have examined KP metabolites without concurrently integrating systemic inflammation and immune-cell redistribution. Moreover, longitudinal studies in antipsychotic-naïve FES remain limited. This is particularly true for designs that can distinguish stable inter-individual differences from within-person fluctuations while rigorously controlling multiplicity in multi-marker settings. Clarifying whether peripheral inflammation, immune-cell phenotypes, and KP balance track cognitive performance across time, and whether cognitive improvement during early treatment is coupled to coordinated immuno-metabolic remodeling, would strengthen mechanistic inference and may identify actionable peripheral signatures.

We enrolled antipsychotic-naïve FES patients and matched healthy controls to profile baseline cognition, inflammation, immune-cell subsets, and KP metabolites and to examine their associations with cognition over 6 months. FES received aripiprazole monotherapy through week 6, followed by naturalistic treatment to month 6. Cognition was assessed with the MCCB, and peripheral blood was analyzed for cytokines/inflammatory markers, immune-cell subsets, and KP metabolites. We hypothesized that higher inflammatory/monocyte burden and a relatively more neurotoxic KP bias would relate to poorer cognition, whereas cognitive improvement would accompany a shift toward a relatively more protective KP balance.

## Methods and materials

### Ethics statement

The study was approved by the Institutional Ethics Committee (EC-KY-2023029) and conducted in accordance with the Declaration of Helsinki and applicable regulations for human subject research. Participants were consecutively recruited between August 2023 and June 2025. Written informed consent was obtained from all participants after a full explanation of study aims, procedures, potential risks, and benefits.

### Sample size calculation

The primary outcome was the MCCB composite score (continuous). Because closed-form sample size calculation for longitudinal LMMs is complex, we used a conservative baseline cross-sectional multiple regression power analysis. Using G*Power (linear multiple regression: Fixed model, R² deviation from zero; two-sided α = 0.05; power = 0.80; number of predictors = 10; assumed overall effect size *f*^2^ = 0.10), the required patient sample size was 118; allowing ~15% attrition, we planned to recruit at least 136 patients. An equal number of healthy controls (n = 136) were enrolled with 1:1 frequency matching by age and sex.

### Participants

This was a single-center prospective longitudinal cohort study of antipsychotic-naïve FES, with an age- and sex-matched healthy controls (HC) reference group assessed at baseline. FES patients were consecutively recruited from the outpatient psychiatry service. Diagnosis was established according to Diagnostic and Statistical Manual of Mental Disorders, Fifth Edition (DSM-5) by at least two qualified psychiatrists using structured/semi-structured interviews and medical records; discrepancies were resolved by consensus. Exclusion criteria: history of severe head trauma (loss of consciousness > 5 min), major neurological disorders (e.g., epilepsy, stroke, dementia) or intellectual disability, inability to complete cognitive testing due to sensory/language impairment, acute infection or antibiotic use within the past 2 weeks, chronic inflammatory/autoimmune disease, malignancy, severe hepatic/renal dysfunction, recent systemic corticosteroids or immunosuppressants, and regular (≥3 days/week) NSAID use within 7 days. Baseline inflammatory screening (temperature, CBC, hs-CRP) was performed; participants with hs-CRP > 10 mg/L or clear evidence of acute infection were excluded. Additional exclusions for FES included substance/medication-induced psychosis, current/past moderate-to-severe substance use disorder, positive urine toxicology suggesting recent illicit substance use (nicotine permitted but quantified), antidepressant use within 30 days before screening, clinically significant risk of harm to self/others or clinical instability, and involuntary hospitalization/treatment. Additional exclusions for HC included any current/past psychiatric diagnosis, substance use disorder or positive urine toxicology, pregnancy or lactation, and severe medical illness.

### Treatment, clinical and cognitive assessments

**Figure 1:**
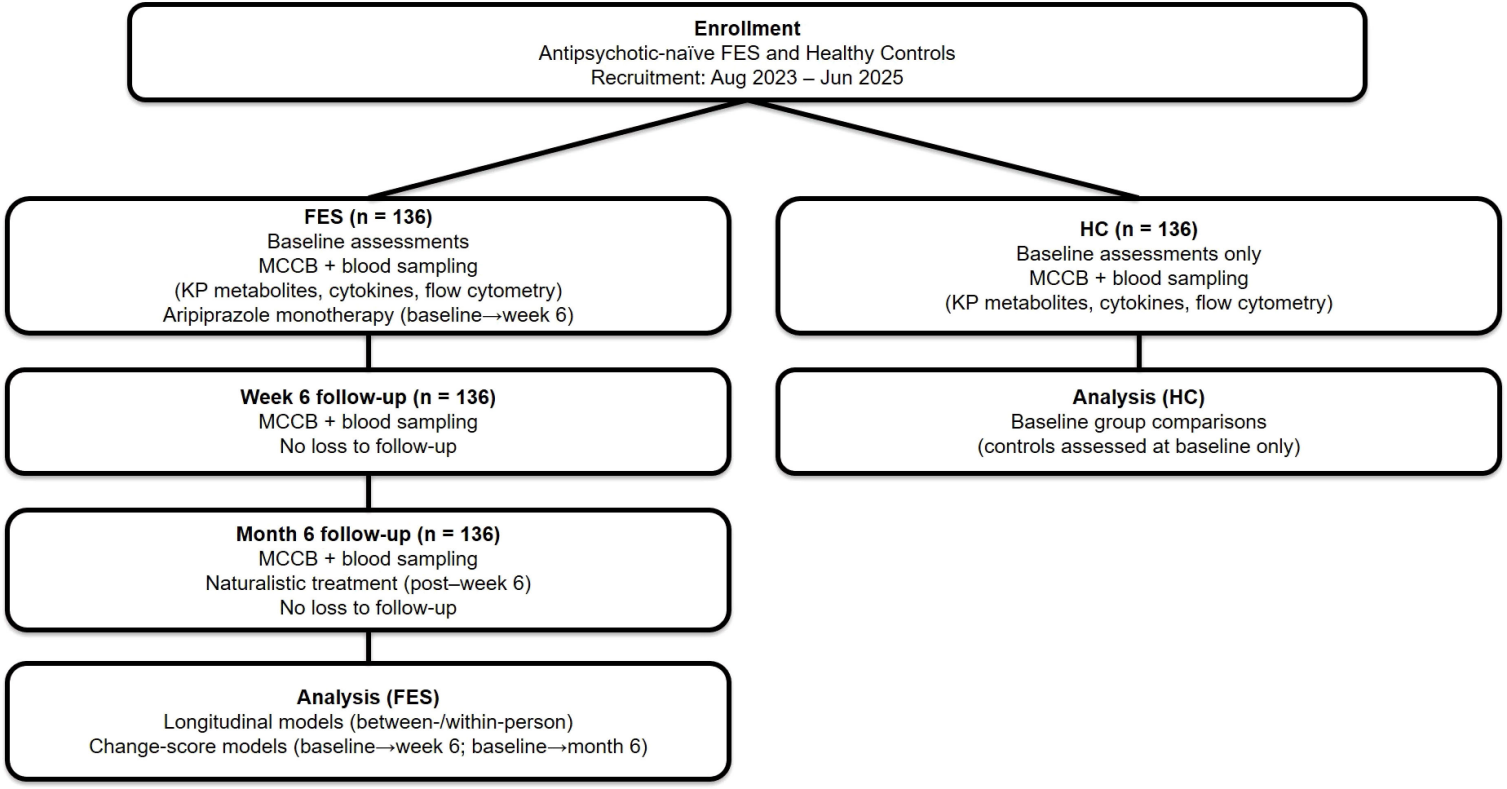
Participant flow of the study cohort. Antipsychotic-naïve first-episode schizophrenia (FES) patients (n = 136) and healthy controls (HC; n = 136) were enrolled between August 2023 and June 2025. HC completed baseline assessments only. FES participants completed baseline, week-6, and month-6 visits, each including cognitive testing (MCCB) and peripheral blood sampling for kynurenine-pathway (KP) metabolites, inflammatory markers, and immune-cell phenotyping. FES received aripiprazole monotherapy from baseline to week 6, followed by naturalistic treatment through month 6. There was no loss to follow-up in the FES cohort.

After baseline assessments, antipsychotic-naïve FES patients received aripiprazole monotherapy until the week-6 visit. After week 6, treatment entered a naturalistic phase; patients who had inadequate clinical response or poor tolerability could be switched to another antipsychotic, whereas others continued aripiprazole. Antipsychotic exposure was standardized as chlorpromazine equivalents (CPZE, mg/day) using established conversion factors (chlorpromazine 100 mg/day equivalent: aripiprazole 7.5 mg, risperidone 2 mg, quetiapine 75 mg, and clozapine 50 mg). Medication adherence during follow-up was assessed using the Brief Adherence Rating Scale (BARS) (20). HC received no intervention and completed baseline procedures only. Psychopathology assessments were administered by trained raters who met predefined inter-rater reliability criteria. Symptom severity at baseline was assessed using the Positive and Negative Syndrome Scale (PANSS) (21) and global functioning with the Global Assessment of Functioning (GAF) (22). Duration of untreated psychosis (DUP) was obtained from patients and corroborated by close relatives when available. Cognitive performance at baseline, week 6, and month 6 was assessed using the MATRICS Consensus Cognitive Battery (MCCB) (23), yielding the composite T-score and seven domain T-scores (processing speed, attention/vigilance, working memory, verbal learning, visual learning, reasoning/problem solving, and social cognition). Participant flow of the study cohort was illustrated in **Figure 1**.

### Blood collection and processing

All participants underwent venipuncture between 07:00 and 10:00 after overnight fasting (≥8 h; water allowed) and avoiding vigorous physical activity for at least 8 h. Blood was collected into K2-EDTA tubes, gently inverted, and processed within 30–60 min. Plasma was separated at 4 °C (1,800 × g for 15 min), aliquoted into low-binding tubes without disturbing the buffy coat, and stored at −80 °C until batch LC–MS/MS and ELISA assays. Laboratory personnel were blinded to group and timepoint during assays and data processing.

### Flow cytometry

K2-EDTA whole blood was processed within 4 h of collection. Surface staining was performed using an antibody panel including CD45, CD3, CD4, CD8, CD19, CD56, CD14, and CD16. After incubation, red blood cells were lysed, samples were washed, and data were acquired on a flow cytometer with compensation based on single-stained controls. Prespecified gating was FSC/SSC → singlets → CD45^+^ leukocytes → lymphocytes/monocytes. Lymphocyte subsets and monocytes were expressed as proportions of CD45^+^ leukocytes; CD16^+^ monocytes were expressed as a proportion of monocytes (% of monocytes).

### Kynurenine pathway metabolites

Plasma TRP, KYN, KYNA, 3-HK, and QA were quantified by LC–MS/MS. Derived ratios included KYN/TRP, KYNA/KYN, QA/KYNA, and KYNA/3-HK. For KYNA/KYN, unit harmonization was performed by converting KYN from μmol/L to nmol/L (×1000) prior to ratio calculation. Sample preparation used 100 μL plasma for protein precipitation with organic solvent containing isotope-labeled internal standards, followed by centrifugation and injection of the supernatant. Detection was performed using electrospray ionization in multiple-reaction monitoring mode with external calibration and internal-standard correction.

### Inflammatory markers

Plasma cytokines were quantified using high-sensitivity Quantikine^®^ ELISA kits (R&D Systems, Bio-Techne; IL-6 HS600C, IFN-γ HSDIF0, TNF-α HSTA00E, IL-1β HSLB00D, IL-10 HS100C, and IL-8 HS800) following the manufacturer’s instructions, with samples assayed in batches to minimize inter-assay variability and avoiding repeated freeze–thaw cycles. hs-CRP was measured in the hospital clinical laboratory using a latex-enhanced immunoturbidimetric assay on an automated chemistry analyzer (Roche cobas c502).

### Statistical analysis

Statistical analyses were performed using GraphPad Prism and Python (statsmodels). Continuous variables are presented as mean ± standard deviation (SD) or median (interquartile range [IQR]), as appropriate, and categorical variables as number (percentage). Baseline comparisons between HC and FES were conducted using independent-samples t tests or Mann–Whitney U tests for continuous variables, and χ² tests or Fisher’s exact tests for categorical variables, as appropriate. Within the FES group, longitudinal changes from baseline to week 6 and month 6 were assessed using repeated-measures ANOVA or Friedman tests according to distributional assumptions, with Holm–Sidak adjustment for *post hoc* pairwise comparisons where applicable. To reduce the influence of right-skewed distributions in regression-based parametric models, hs-CRP, IL-6, and KYN/TRP were natural-log transformed when entered as continuous predictors or outcomes. To explore whether changes in KP metabolites might reflect direct pharmacological effects of antipsychotic treatment, we performed exploratory time-matched analyses using CPZE (mg/day) at each follow-up visit. CPZE represented the average daily dose at the corresponding visit rather than cumulative exposure. Associations between CPZE and changes in KP metabolites from baseline were examined using Spearman’s rank correlation. As a sensitivity analysis, changes in KP metabolites were also compared between patients who remained on aripiprazole and those who switched to another antipsychotic during follow-up, with false discovery rate (FDR) correction applied across KP outcomes. Cross-sectional associations at each time point were assessed using Spearman’s rank correlation. These included correlations between MCCB composite T scores and biomarkers, as well as between inflammatory markers and KP metabolites. To account for multiple testing, *P* values were adjusted using the Benjamini–Hochberg FDR procedure, and FDR-adjusted q values are reported. For longitudinal modeling within the FES group, MCCB composite T score was analyzed across baseline, week 6, and month 6 using linear mixed-effects models with a subject-specific random intercept and time specified as a categorical fixed effect. To distinguish stable inter-individual differences from intra-individual fluctuations, each biomarker was decomposed into a between-person component (participant-specific mean) and a within-person component (time-specific deviation from that mean). Models were adjusted for sex, age, education, BMI, smoking, drinking, DUP, baseline PANSS total score, and time-varying CPZE. Multiplicity across biomarkers was controlled using the Benjamini–Hochberg FDR procedure. Change-score analyses were performed to test whether cognitive improvement tracked immuno-metabolic shifts over follow-up. For each interval (baseline to week 6; baseline to month 6), change in MCCB composite score (Δ_MCCB_) was regressed on the corresponding change in biomarker (Δ _biomarker_), adjusting for baseline MCCB, baseline biomarker level, sex, age, education, DUP, baseline PANSS total score, and CPZE at the corresponding follow-up visit. Two-sided *P* values and FDR-adjusted q values are reported, with statistical significance defined as q < 0.05. Finally, exploratory baseline mediation analyses were conducted within the FES group to examine whether KP imbalance statistically linked peripheral inflammation to cognitive performance. In these models, baseline MCCB composite T score was specified as the outcome, baseline KYNA/3-HK as the mediator, and each baseline inflammatory marker was tested separately as the predictor. Models were adjusted for sex, age, education, BMI, smoking, drinking, DUP, and baseline PANSS total score. Predictors of interest showing right-skew (hs-CRP and IL-6) were natural-log transformed before modeling, and the focal predictor and mediator were z-standardized to facilitate comparison of effect sizes. Mediation was evaluated using ordinary least squares regression with bootstrap estimation of indirect effects (2,000 resamples), and total, direct, and indirect effects with 95% bootstrap confidence intervals are reported.

## Results

### Baseline characteristics

There was no participant attrition in the FES cohort at week 6 or month 6. Baseline demographic characteristics were comparable between HC and FES (**Table 1**), with no significant between-group differences in age, sex, years of education, BMI, current smoking status, or current drinking status (all *P* > 0.05). In contrast, global functioning was significantly lower in FES, reflected by reduced GAF scores (median 59.0 *vs* 73.0; *P <* 0.001). Clinical characteristics were summarized for FES only: DUP was 15.0 weeks, and symptom severity was captured by PANSS scores (positive 21.0, negative 22.0, general 46.0, total 89.5). At week 6, all FES participants received aripiprazole monotherapy (median 15.0 mg/day) with mean CPZE of 148.3 ± 44.9 mg/day; at month 6, CPZE increased to 229.1 ± 121.6 mg/day, with aripiprazole remaining the most common treatment [aripiprazole 113/136 (83.1%), quetiapine 10/136 (7.4%), risperidone 9/136 (6.6%), and clozapine 4/136 (2.9%)]. Mean BARS adherence was 87.6% at week 6 and 81.4% at month 6, indicating generally good adherence during follow-up.

**Table 1:** Baseline characteristics of healthy controls (HC) and first-episode schizophrenia (FES) patients.

Variable	HC (n = 136)	FES (n = 136)	*P* value
Age, years	30.00 [25.00, 35.00]	29.50 [24.00, 33.00]	0.689
Male, n (%)	74 (54.4%)	85(62.5%)	0.219
Education, years	13.00 [12.00, 16.00]	13.00 [12.00, 16.00]	0.501
Body mass index, kg/m²	22.86 ± 3.32	22.50 ± 3.38	0.351
Current smoking, n (%)	83 (61.0%)	96 (70.6%)	0.125
Current drinking, n (%)	62 (45.6%)	52 (38.2%)	0.276
GAF score	73.00 [70.00, 76.00]	59.00 [50.00, 70.00]	< 0.001
Duration of untreated psychosis (DUP), weeks	–	15.00 [8.00, 28.00]	
PANSS positive	–	21.00 [18.00, 23.00]	
PANSS negative	–	22.00 [19.00, 25.00]	
PANSS general	–	46.00 [41.00, 51.00]	
PANSS total	–	89.50 [81.00, 98.00]	
Aripiprazole dose at week 6, mg/day	–	15.00 [10.00, 20.00]	
Antipsychotic compound at month 6			
Aripiprazole	–	113	
Quetiapine	–	10	
Risperidone	–	9	
Clozapine	–	4	
Chlorpromazine equivalents (CPZE), mg/day			
At week 6	–	148.3 ± 44.9	
At month 6	–	229.1 ± 121.6	

PANSS positive and negative syndrome scale.

### Baseline cognitive deficits and longitudinal cognitive change

**Figure 2:**
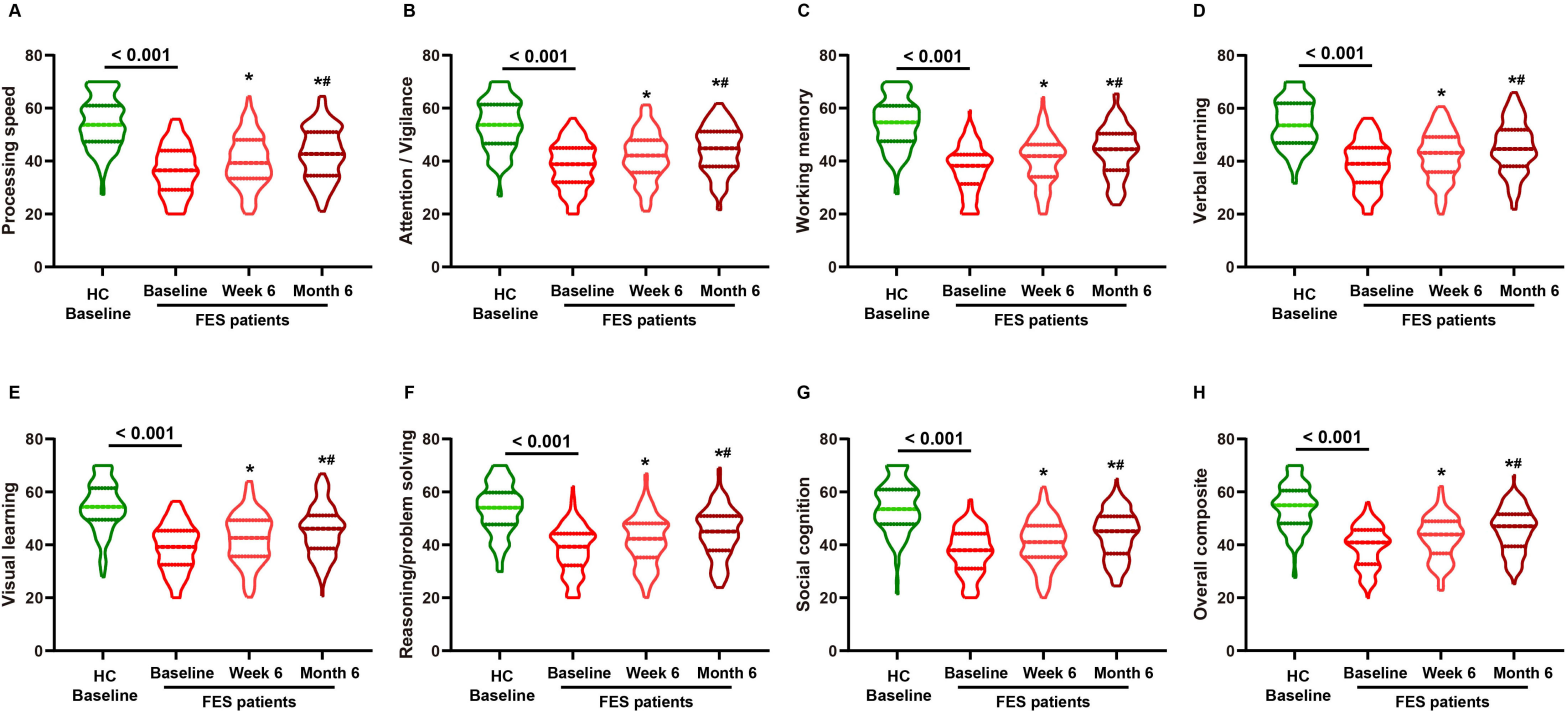
Longitudinal changes in MCCB domain and composite T-scores in FES patients, with baseline comparison against healthy controls. Notes: Violin plots display MATRICS Consensus Cognitive Battery (MCCB) T-scores for healthy controls (HC; baseline only) and first-episode schizophrenia (FES) assessed at baseline, week 6, and month 6. Panels show **(A)** Processing speed, **(B)** Attention/Vigilance, **(C)** Working memory, **(D)** Verbal learning, **(E)** Visual learning, **(F)** Reasoning/Problem solving, **(G)** Social cognition, and **(H)** Overall composite score. Higher T-scores indicate better cognitive performance. *P < 0.05 *vs.* FES baseline; #*P <* 0.05 *vs.* FES week 6.

As shown in **Figure 2**, FES patients exhibited marked global cognitive impairment at baseline, with substantially lower MCCB composite T-scores than HC (median 40.90 *vs* 54.95; *P <* 0.001). All seven MCCB domains were also significantly reduced in FES (all *P <* 0.001). During follow-up, MCCB composite performance improved over time in FES (*P <* 0.05), with stepwise gains by week 6 and further improvement by month 6; domain-level T-scores showed parallel increases (all *P <* 0.05).

### Kynurenine pathway metabolites over follow-up

KP metabolites were summarized in **Table 2**. At baseline, FES showed a pattern consistent with KP imbalance, including lower TRP and a higher KYN/TRP ratio (both *P <* 0.001). Downstream metabolites suggested a shift toward a relatively more neurotoxic profile, with reduced KYNA and KYNA/KYN and increased 3-HK and QA (all *P <* 0.001). Accordingly, QA/KYNA was elevated and KYNA/3-HK reduced in FES (both *P <* 0.001). Over follow-up, KP markers changed significantly within the FES group (all *P <* 0.05), indicating partial normalization over time, characterized by increases in TRP and KYNA and decreases in KYN/TRP, 3-HK, QA, and QA/KYNA. In exploratory dose–response analyses, no significant associations were observed between CPZE and changes in KP metabolites at either week 6 or month 6 after FDR correction (all q > 0.05). For change from baseline to month 6, sensitivity analyses showed no significant differences in KP changes between patients who remained on aripiprazole and those who switched to other antipsychotics (all q > 0.05, **Supplementary Table 1**).

**Table 2:** Baseline between-group differences and longitudinal changes in kynurenine pathway metabolite levels in healthy controls (HC) and first-episode schizophrenia (FES) participants.

Marker	HC	FES patients			Between-group *P* (baseline)	Longitudinal *P* (FES)
		Baseline	Week 6	Month 6		
TRP (μmol/L)	62.8 [54.35, 76.65]	57.2 [46.43, 69.13]	60.35 [53.98, 67.02] ^*^	62.49 [59.37, 65.19] *^#^	< 0.001	< 0.001
KYN (μmol/L)	2.3 [1.9, 2.7]	2.3 [1.8, 2.7]	2.3 [1.8, 2.7]	2.279 [2.0, 2.5]	> 0.05	> 0.05
KYN/TRP	35.08 [28.52, 42.63]	38.57 [30.85, 49.75]	37.96 [32.1, 42.7] ^*^	36.48 [33.85, 38.85] *^#^	< 0.001	< 0.001
KYNA (nmol/L)	60.45 [49.70, 70.15]	44.95 [37.95, 52.1]	51.17 [47.18, 55.78] ^*^	55.86 [53.36, 57.83] *^#^	< 0.001	< 0.001
KYNA/KYN	0.026 [0.021, 0.033]	0.020 [0.016, 0.025]	0.023 [0.020, 0.026] ^*^	0.024 [0.023, 0.026] *^#^	< 0.001	< 0.001
3-HK (nmol/L)	54.60 [43.78, 64.13]	64.50 [54.90, 75.35]	60.44 [54.31, 66.71] ^*^	57.33 [54.32, 60.49] *^#^	< 0.001	< 0.001
QA (nmol/L)	397.8 [308.5, 471.5]	469.2 [341.3, 589.4]	442.0 [359.7, 509.4] ^*^	418.0 [380.1, 453.8] *^#^	< 0.001	< 0.001
QA/KYNA	6.613 [5.303, 8.05]	10.46 [7.715, 13.22]	8.553 [7.25, 10.18] ^*^	7.515 [6.837, 8.157] *^#^	< 0.001	< 0.001
KYNA/3-HK	1.116 [0.873, 1.369]	0.709 [0.561, 0.860]	0.860[0.741, 0.950] ^*^	0.974 [0.904, 1.033] *^#^	< 0.001	< 0.001

Data are presented as median [interquartile range]. Between-group testing was performed only at baseline (HC baseline *vs* FES baseline) using the Mann–Whitney U test (*P* value shown above the bracket). All other comparisons reflect within-FES longitudinal analyses using repeated-measures one-way ANOVA with Greenhouse–Geisser correction, followed by Holm–Sidak *post hoc* tests. *P < 0.05 *vs* FES baseline; #*P <* 0.05 *vs* FES week 6.

### Systemic inflammatory profiles and immune cell subset distribution over follow-up

**Figure 3:**
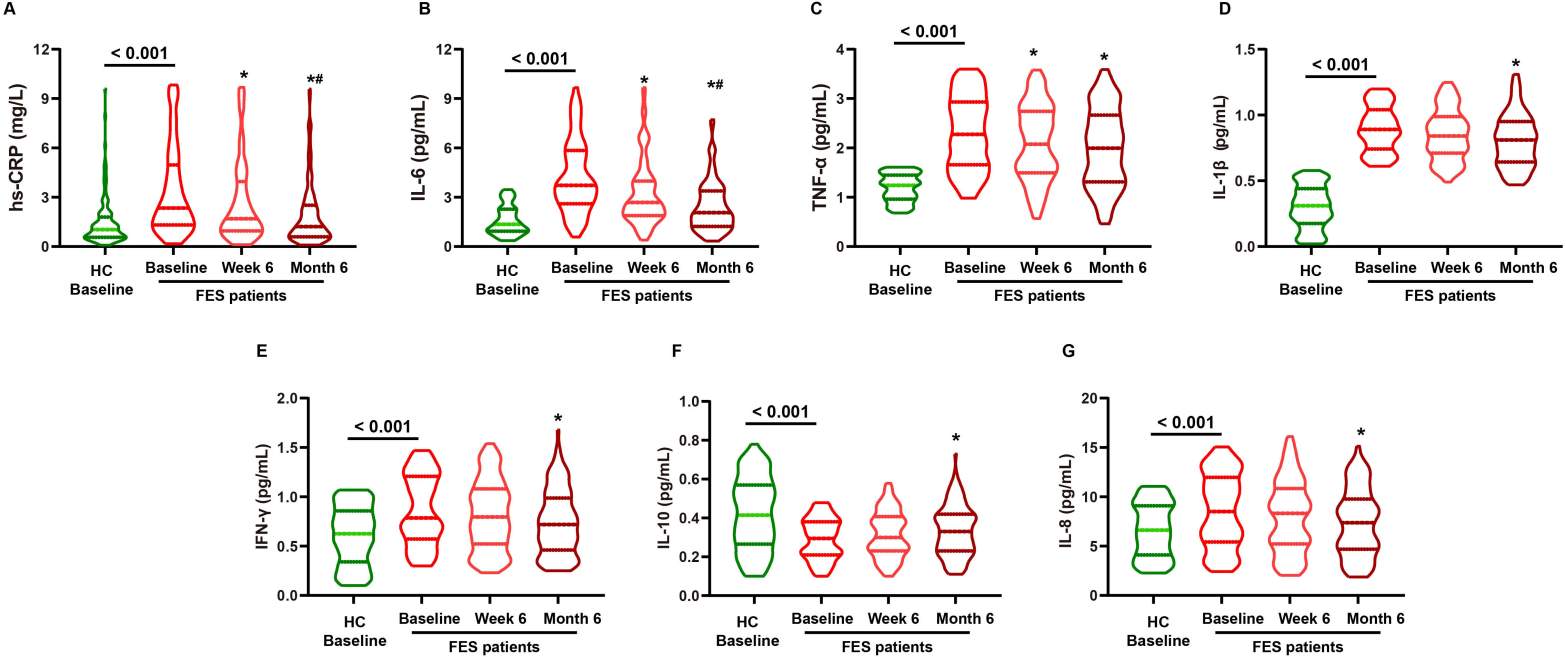
Longitudinal changes in inflammatory markers in FES patients, with baseline comparison to healthy controls. Notes: Violin plots display inflammatory markers for healthy controls (HC; baseline only) and first-episode schizophrenia (FES) assessed at baseline, week 6, and month 6. Panels show **(A)** hs-CRP, **(B)** IL-6, **(C)** TNF-α, **(D)** IL-1β, **(E)** IFN-γ, **(F)** IL-10, and **(G)** IL-8. *P < 0.05 *vs.* FES baseline; #*P <* 0.05 *vs.* FES week 6.

As shown in **Figure 3**, FES patients demonstrated higher systemic inflammatory burden at baseline than HC (all *P <* 0.001), including higher hs-CRP, IL-6, TNF-α, IL-1β, IFN-γ, and IL-8, alongside lower IL-10. Over follow-up, inflammatory markers changed significantly within FES, with decreases in hs-CRP and pro-inflammatory cytokines and a gradual increase in IL-10, consistent with progressive normalization by week 6 and further improvement by month 6. As shown in **Table 3**, FES also exhibited marked immune-cell redistribution at baseline. Relative to HC, FES showed lower proportions of CD3^+^ T cells, CD4^+^ T cells and CD8^+^ T cells, together with a lower CD4/CD8 ratio (all *P <* 0.001). In contrast, CD14^+^ monocytes were elevated and the monocyte compartment shifted toward a CD16^+^ phenotype (both *P <* 0.001). NK (CD56^+^) cells were also increased (*P <* 0.001), whereas CD19^+^ B cells showed no significant difference (*P =* 0.372). Longitudinally, immune-cell distributions in FES moved toward the HC baseline profile, with increasing T-cell proportions and declining monocyte proportions.

**Table 3:** Baseline between-group differences and longitudinal changes in immune cell subset distribution in healthy controls (HC) and first-episode schizophrenia (FES) participants.

Marker	HC	FES patients			Between-group *P* (baseline)	Longitudinal *P* (FES)
		Baseline	Week 6	Month 6		
CD4^+^ T cells (% of CD45)	35.59 [33.94, 37.09]	28.78 [27.22, 30.44]	31.09 [29.57, 33.10] ^*^	32.91 [30.75, 34.68] *^#^	< 0.001	< 0.001
CD8^+^ T cells (% of CD45)	22.71 [21.59, 23.64]	21.61 [20.27, 23.46]	22.04 [20.10, 23.71] ^*^	22.00 [20.15, 23.53] ^*^	< 0.001	< 0.001
CD3^+^ T cells (% of CD45)	64.94 [63.39, 66.91]	57.85 [56.03, 59.66]	60.59 [58.47, 62.45] ^*^	62.18 [59.62, 64.48] *^#^	< 0.001	< 0.001
CD19^+^ B cells (% of CD45)	11.94 [10.66, 13.10]	11.53 [10.54, 13.43]	10.95 [9.84, 12.57] ^*^	10.39 [9.25, 12.14] *^#^	< 0.001	< 0.001
NK (CD56^+^) cells (% of CD45)	9.64 [9.01, 10.81]	8.01 [7.13, 9.16]	8.21 [7.34, 9.43] ^*^	8.46 [7.34, 9.59] *^#^	< 0.001	< 0.001
CD14^+^ monocytes (% of CD45)	7.80 [6.94, 8.77]	10.80 [9.56, 11.92]	9.36 [8.14, 10.46] ^*^	8.43 [7.49, 9.72] *^#^	< 0.001	< 0.001
Other leukocytes (% of CD45)	4.95 [4.24, 5.94]	11.29 [9.97, 12.39]	10.44 [9.16, 11.63] ^*^	10.02 [8.66, 11.18] *^#^	< 0.001	< 0.001
CD16^+^ monocytes (% of monocytes)	19.68 [17.87, 21.26]	24.67 [22.97, 26.54]	23.56 [21.79, 25.34] ^*^	22.67 [20.73, 24.81] *^#^	< 0.001	< 0.001
CD4/CD8 ratio	1.56 [1.44, 1.70]	1.33 [1.18, 1.49]	1.41 [1.28, 1.57] ^*^	1.48 [1.33, 1.68] *^#^	< 0.001	< 0.001

Data are presented as median [interquartile range]. Between-group testing was performed only at baseline (HC baseline *vs* FES baseline) using the Mann–Whitney U test (*P* value shown above the bracket). All other comparisons reflect within-FES longitudinal analyses using repeated-measures one-way ANOVA with Greenhouse–Geisser correction, followed by Holm–Sidak *post hoc* tests. *P < 0.05 *vs* FES baseline; #*P <* 0.05 *vs* FES week 6.

### Correlations between cognition and immune–kynurenine biomarkers at baseline, week 6, and month 6

**Figure 4:**
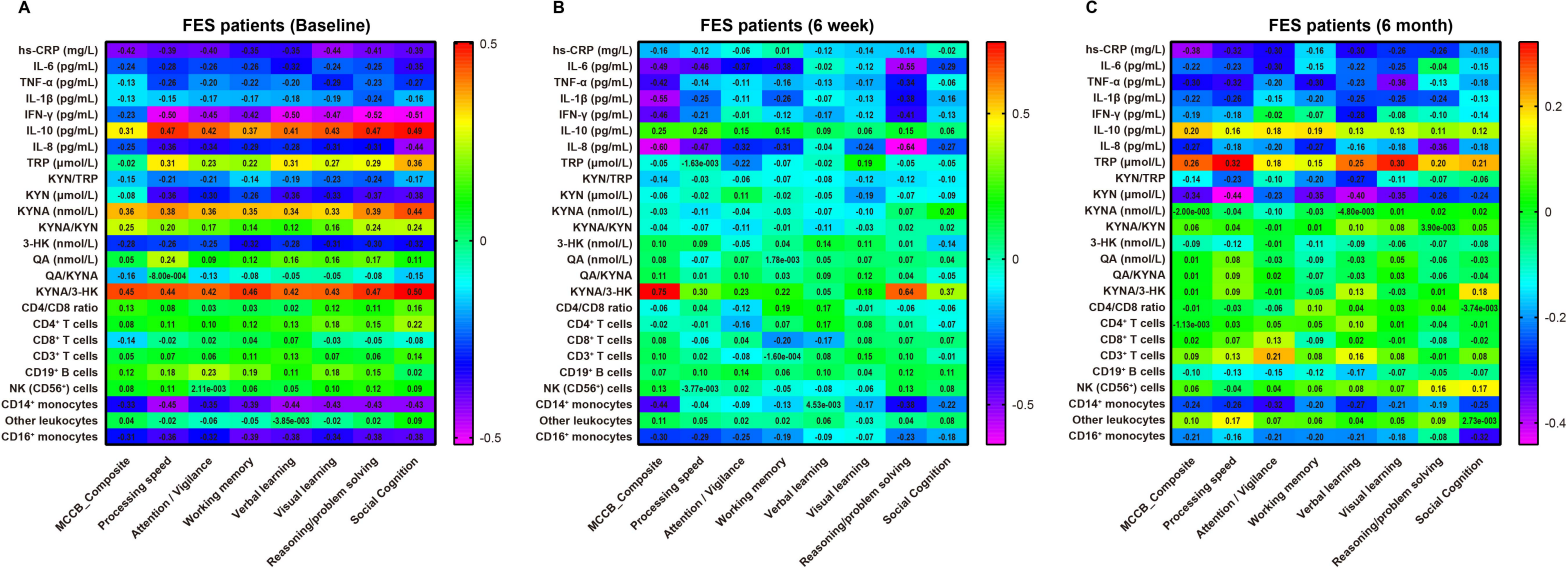
Spearman correlation matrices between MCCB score and immuno-metabolic markers at baseline, week 6, and month 6 in FES. Panels show cross-sectional Spearman’s rank correlations (ρ) within the FES group at **(A)** baseline, **(B)** week 6, and **(C)** month 6. Variables include the MCCB composite T score and peripheral biomarkers spanning systemic inflammation (hs-CRP and cytokines), kynurenine pathway (KP) metabolites/ratios, and major immune-cell subsets/monocyte indices. Color intensity represents the correlation coefficient (ρ).

Cross-sectional Spearman analyses showed that better cognitive performance co-occurred with a relatively more protective KP balance and a lower inflammatory and monocyte burden. At baseline (**Figure 4A**), MCCB composite score was positively correlated with KYNA/3-HK (ρ = 0.447, q < 0.001) and inversely correlated with hs-CRP (ρ = −0.417, q < 0.001), CD14^+^ monocytes (ρ = −0.327, q < 0.001), and CD16^+^ monocytes (% of monocytes) (ρ = −0.307, q < 0.001). At week 6 (**Figure 4B**), these associations became more pronounced: MCCB composite remained strongly positively correlated with KYNA/3-HK (ρ = 0.746, q < 0.001) and showed robust inverse correlations with multiple pro-inflammatory markers and monocyte measures, including IL-8 (ρ = −0.602, q < 0.001), IL-1β (ρ = −0.547, q < 0.001), IL-6 (ρ = −0.487, q < 0.001), IFN-γ (ρ = −0.462, q < 0.001), TNF-α (ρ = −0.423, q < 0.001), CD14^+^ monocytes (ρ = −0.439, q < 0.001), and CD16^+^ monocytes (% of monocytes) (ρ = −0.300, q = 0.003). IL-10 showed a modest positive correlation (ρ = 0.246, q = 0.025). By month 6 (**Figure 4C**), the correlation pattern narrowed and primarily reflected inflammatory burden and TRP metabolism: MCCB composite was inversely correlated with hs-CRP (ρ = −0.384, q < 0.001) and KYN/TRP (ρ = −0.339, q = 0.001), positively correlated with TRP (ρ = 0.265, q = 0.015), and remained inversely associated with CD14^+^ monocytes (ρ = −0.243, q = 0.024). Cross-sectional correlations between inflammatory markers and KP metabolites are summarized in **Supplementary Table 2**. Baseline showed the largest number of significant inflammations–KP associations. The strongest included a positive correlation between IL-10 and KYNA/3-HK (ρ = 0.682, q *<* 0.001) and a strong negative correlation between IFN-γ and KYNA/3-HK (ρ = -0.611, q *<* 0.001). In addition, IFN-γ was negatively correlated with KYNA (ρ = -0.521, q *<* 0.001), and IL-8 was negatively correlated with KYNA/3-HK (ρ = -0.476, q *<* 0.001). hs-CRP was positively correlated with KYN/TRP (ρ = 0.308, q *=* 0.001) and negatively correlated with KYNA (ρ = -0.384, q *<* 0.001) and KYNA/3-HK (ρ = -0.378, q *<* 0.001), further supporting an association between greater inflammatory burden and a less favorable KP profile. At week 6, the remaining significant associations were concentrated almost exclusively on KYNA/3-HK, including inverse correlations with IFN-γ, IL-8, IL-6, and TNF-α, together with a positive correlation with IL-10. The strongest week-6 associations were IL-8 versus KYNA/3-HK (ρ = -0.409, q *<* 0.001) and IFN-γ versus KYNA/3-HK (ρ = -0.347, q *=* 0.001). By month 6, only one association remained significant after FDR correction, namely IL-1β with KYN/TRP (ρ = 0.321, q = 0.009), indicating a marked weakening of cross-sectional inflammation–KP coupling over follow-up.

### Longitudinal mixed-effects associations of immune–kynurenine biomarkers with MCCB composite score

In longitudinal mixed-effects models that decomposed each biomarker into between-person (person-mean) and within-person (time-varying deviation) components, associations with MCCB composite score were driven predominantly by between-person effects (**Table 4**). Higher systemic inflammatory burden was consistently associated with lower cognition, including hs-CRP (β/SD = −2.32, q *=* 7.8E-6), IL-6 (β/SD = −3.37, q *=* 2.7E-12), TNF-α (β/SD = −2.87, q *=* 8.4E-7), IL-1β (β/SD = −3.16, q *=* 9.2E-12), IFN-γ (β/SD = −3.46, q *=* 3.4E-16), and IL-8 (β/SD = −3.80, q *=* 1.1E-13). In contrast, IL-10 was positively associated with cognition (β/SD = +3.47, q *=* 5.7E-13). KP indices showed convergent between-person relationships: higher KYNA (β/SD = +2.52, q *=* 1.7E-6), KYNA/KYN (β/SD = +1.92, q *=* 0.001), and particularly KYNA/3-HK (β/SD = +4.82, q *=* 2.5E-25) were associated with higher MCCB scores, whereas 3-HK was inversely associated (β/SD = −2.33, q *=* 2.8E-5). KYN and KYN/TRP also showed negative associations with cognition (KYN: β/SD = −1.29, q *=* 0.035; KYN/TRP: β/SD = −1.44, q *=* 0.034), while TRP was not significant. For immune-cell subsets, higher CD14^+^ monocytes (β/SD = −3.67, q *=* 5.1E-12) and higher CD16^+^ monocytes (% of monocytes; β/SD = −2.74, q *=* 1.3E-8) were strongly associated with lower MCCB scores. At the within-person level, several biomarkers showed nominal *P* values, but none remained significant after BH–FDR correction, indicating that the strongest evidence supported stable inter-individual immuno-metabolic differences rather than concurrent within-person covariation with cognition.

**Table 4:** Longitudinal mixed-effects associations of immune–kynurenine biomarkers with MCCB composite score.

Biomarker	Between-person level			Within-person		
	β/SD	*P*	q_FDR	β/SD	*P*	q_FDR
hs-CRP (mg/L)	-2.32	**3.4E-06**	**7.8E-06**	-0.35	0.226	0.488
IL-6 (pg/mL)	-3.37	**5.3E-13**	**2.7E-12**	-0.51	0.134	0.488
TNF-α (pg/mL)	-2.87	**3.0E-07**	**8.4E-07**	-0.46	0.185	0.488
IL-1β (pg/mL)	-3.16	**2.6E-12**	**9.2E-12**	-0.47	0.182	0.488
IFN-γ (pg/mL)	-3.46	**2.8E-17**	**3.4E-16**	-0.64	**0.039**	0.486
IL-10 (pg/mL)	3.47	**9.2E-14**	**5.7E-13**	-0.50	0.096	0.488
IL-8 (pg/mL)	-3.80	**1.3E-14**	**1.1E-13**	-0.76	0.061	0.488
TRP (μmol/L)	0.42	0.455	0.494	-0.06	0.845	0.918
KYN (μmol/L)	-1.29	**0.021**	**0.035**	-0.31	0.338	0.563
KYN/TRP	-1.44	**0.019**	**0.034**	0.26	0.337	0.563
KYNA (nmol/L)	2.52	**6.8E-07**	**1.7E-06**	0.35	0.224	0.488
KYNA/KYN	1.92	**4.5E-04**	**0.001**	-0.31	0.316	0.563
3-HK (nmol/L)	-2.33	**1.3E-05**	**2.8E-05**	-0.33	0.234	0.488
QA (nmol/L)	1.03	**0.044**	0.068	-0.23	0.399	0.623
QA/KYNA	-0.57	0.285	0.340	0.13	0.762	0.907
KYNA/3-HK	4.82	**9.9E-27**	**2.5E-25**	0.70	**0.022**	0.486
CD4/CD8 ratio	0.45	0.405	0.460	0.06	0.815	0.918
CD4^+^ T cells	0.78	0.159	0.209	0.14	0.643	0.846
CD8^+^ T cells	-0.24	0.646	0.673	-0.03	0.929	0.930
CD3^+^ T cells	0.73	0.185	0.231	0.10	0.720	0.900
CD14^+^ monocytes	-3.67	**1.2E-12**	**5.1E-12**	-0.48	0.147	0.488
NK (CD56^+^) cells	0.82	0.107	0.148	-0.21	0.599	0.832
CD19^+^ B cells	0.01	0.975	0.975	0.03	0.930	0.930
Other leukocytes	0.99	0.066	0.097	-0.17	0.532	0.782
CD16^+^ monocytes (% of monocytes)	-2.74	**4.1E-09**	**1.3E-08**	-0.39	0.184	0.488

Values are presented as standardized effect sizes (β/SD), indicating the change in MCCB composite T score (higher scores reflect better cognition) associated with a 1–SD increase in each biomarker. A person-mean decomposition approach was used to separate between-person effects (inter-individual differences in an individual’s long-term mean biomarker level) from within-person effects (time-specific deviations from an individual’ s own mean level). Models were adjusted for sex, age (years), education (years), duration of untreated psychosis (DUP, weeks), baseline PANSS total score, and time (baseline, week 6, month 6), as well as time-varying antipsychotic exposure expressed as chlorpromazine equivalents (CPZE). *P* values are two-sided. Bold values indicate statistically significant. Q_FDR denotes Benjamini–Hochberg false discovery rate–adjusted q values; statistical significance was defined as q_FDR < 0.05. MCCB, MATRICS Consensus Cognitive Battery; TRP, tryptophan; KYN, kynurenine; KYNA, kynurenic acid; 3-HK, 3-hydroxykynurenine; QA, quinolinic acid; hs-CRP, high-sensitivity C-reactive protein.

### Change-score analysis of cognitive improvement and immuno-metabolic shifts

Change-score models examined whether interval changes in biomarkers tracked the corresponding change in MCCB composite score (Δ _MCCB_) from baseline to week 6 and from baseline to month 6 (**Table 5**). After BH-FDR correction, significant associations were confined to KP remodeling, whereas changes in cytokines and immune-cell fractions did not survive correction in either interval. From baseline to week 6, Δ _MCCB_ was significantly associated with increases in KYNA (β/SD = +2.59, q *=* 5.15E-5) and KYNA/KYN (β/SD = +1.77, q *=* 0.010), and inversely associated with 3-HK change (β/SD = −2.13, q *=* 0.001). From baseline to month 6, these KP signals persisted and strengthened: Δ _MCCB_ was positively associated with Δ _KYNA_ (β/SD = +2.99, q *=* 4.62E-4), Δ _KYNA/KYN_ (β/SD = +2.23, q *=* 0.021), and Δ _KYNA/3-HK_ (β/SD = +3.90, q *=* 1.45E-8), while remaining inversely associated with Δ _3-HK_ (β/SD = −2.17, q *=* 0.015). In contrast, change measures for hs-CRP, pro-inflammatory cytokines, IL-10, and immune-cell subsets (including CD14^+^ and CD16^+^ monocyte measures) did not remain significant after BH-FDR correction. These findings suggest that KP rebalancing, rather than broad inflammatory or immune-cell redistribution, was the most reproducible immuno-metabolic correlate of cognitive improvement during follow-up.

**Table 5:** Change-score analysis of cognitive improvement and immuno-metabolic shifts.

Biomarker	Baseline→Week6			Baseline→Month6		
	β/SD (Delta)	*P*	q_FDR	β/SD (Delta)	*P*	q_FDR
hs-CRP (mg/L)	1.35	**0.025**	0.126	-0.30	0.713	0.872
IL-6 (pg/mL)	-0.77	0.205	0.412	0.32	0.688	0.872
TNF-α (pg/mL)	-0.68	0.214	0.412	-0.35	0.664	0.872
IL-1β (pg/mL)	-1.29	0.052	0.217	-0.04	0.959	0.996
IFN-γ (pg/mL)	-0.02	0.975	0.975	0.85	0.208	0.603
IL-10 (pg/mL)	-1.44	**0.017**	0.108	-0.99	0.117	0.487
IL-8 (pg/mL)	-0.10	0.886	0.967	0.71	0.320	0.728
TRP (μmol/L)	-0.91	0.150	0.390	-0.24	0.733	0.872
KYN (μmol/L)	0.12	0.849	0.967	0.43	0.579	0.854
KYN/TRP	0.75	0.214	0.412	0.64	0.392	0.754
KYNA (nmol/L)	2.59	**2.06E-06**	**5.15E-05**	2.99	**3.69E-05**	**4.62E-04**
KYNA/KYN	1.77	**0.001**	**0.010**	2.23	**0.003**	**0.021**
3-HK (nmol/L)	-2.13	**1.18E-04**	**0.001**	-2.17	**0.002**	**0.015**
QA (nmol/L)	1.13	0.083	0.297	-0.97	0.182	0.603
QA/KYNA	0.23	0.742	0.928	0.43	0.581	0.854
KYNA/3-HK	0.41	0.478	0.702	3.90	**5.80E-10**	**1.45E-08**
CD4/CD8 ratio	-1.07	0.113	0.354	-0.69	0.310	0.728
CD4^+^ T cells	-0.69	0.291	0.519	-0.73	0.374	0.754
CD8^+^ T cells	0.94	0.156	0.390	0.45	0.523	0.854
CD3^+^ T cells	0.08	0.905	0.967	-0.62	0.433	0.773
CD14^+^ monocytes	-0.49	0.525	0.728	1.20	0.085	0.425
NK (CD56^+^) cells	0.55	0.392	0.612	0.17	0.817	0.910
CD19^+^ B cells	0.05	0.929	0.967	0.00	0.996	0.996
Other leukocytes	-0.51	0.380	0.612	-0.16	0.837	0.910
CD16^+^ monocytes (% of monocytes)	0.37	0.578	0.760	0.88	0.217	0.603

The outcome was cognitive improvement, defined as ΔMCCB composite T score (follow-up minus baseline; positive values indicate improvement). Separate models were fitted for baseline→week 6 and baseline→month 6 intervals. β/SD (Delta) denotes the standardized regression coefficient for the change in each biomarker (Δ _biomarker_), corresponding to the expected change in ΔMCCB per 1–SD increase in Δ _biomarker_. Models were adjusted for baseline MCCB composite T score, baseline biomarker level, sex, age (years), education (years), duration of untreated psychosis (DUP, weeks), baseline PANSS total score, and interval-specific antipsychotic exposure expressed as chlorpromazine equivalents (CPZE) (week 6 CPZE for the baseline→week 6 model; month 6 CPZE for the baseline→month 6 model). P values are two-sided. q_FDR indicates Benjamini–Hochberg false discovery rate–adjusted q values; statistical significance was defined as q_FDR < 0.05. Bold values indicate statistically significant. MCCB, MATRICS Consensus Cognitive Battery; KP, kynurenine pathway; TRP, tryptophan; KYN, kynurenine; KYNA, kynurenic acid; 3-HK, 3-hydroxykynurenine; QA, quinolinic acid; hs-CRP, high-sensitivity C-reactive protein.

### Baseline mediation analyses

After adjustment for sex, age, education, BMI, smoking, drinking, DUP, and baseline PANSS total score, significant bootstrap-based indirect effects were observed for IFN-γ (indirect β = -0.285, 95% CI -0.408 to -0.162, bootstrap *P* < 0.001), hs-CRP (indirect β = -0.129, 95% CI -0.226 to -0.055, bootstrap *P* < 0.001), IL-6 (indirect β = -0.103, 95% CI -0.197 to -0.024, bootstrap *P* = 0.007), IL-8 (indirect β = -0.196, 95% CI -0.315 to -0.096, bootstrap *P* < 0.001), and IL-10 (indirect β = 0.259, 95% CI 0.123 to 0.377, bootstrap *P* < 0.001). For IFN-γ, IL-6, IL-8, and IL-10, the direct effects were attenuated to non-significance after inclusion of KYNA/3-HK, whereas hs-CRP retained a significant direct effect, consistent with partial mediation. Overall, these findings support an inflammation–KP–cognition link in FES, although the observational design does not permit causal inference.

## Discussion

In this longitudinal study of antipsychotic-naïve FES, patients exhibited marked baseline cognitive impairment with stepwise improvement through week 6 and month 6, consistent with early cognitive gains during initial treatment and stabilization. In FDR-controlled mixed-effects models, lower MCCB composite scores were robustly associated at the between-person level with greater inflammatory burden and monocyte-related indices, whereas IL-10 and KP indices reflecting a more “protective” balance (KYNA, KYNA/KYN, KYNA/3-HK) tracked better cognition. In change-score analyses, cognitive improvement was most consistently coupled to KP remodeling—positive associations with Δ_KYNA_, Δ_KYNA/KYN_, and Δ_KYNA/3-HK_ (month 6), alongside an inverse association with Δ3-HK—whereas changes in cytokines and immune-cell fractions did not remain robust after FDR correction. Collectively, these findings implicate KP dynamics as a reproducible immuno-metabolic correlate of cognitive change during early follow-up.

The pronounced baseline MCCB impairment in antipsychotic-naïve FES aligns with evidence that broad cognitive deficits are already present at first episode (24). The stepwise improvement by week 6 and month 6 is also consistent with longitudinal MCCB studies in FES, where modest gains are often observed with early treatment and clinical stabilization (25). Such improvement likely reflects a combination of symptom reduction, reduced interference from acute psychotic symptoms during cognitive testing, repeated-testing effects, and potential medication-related influences. Notably, biomarker–cognition associations persisted at the between-person level despite overall cognitive improvement.

At baseline, FES demonstrated elevated systemic inflammation (hs-CRP and multiple pro-inflammatory cytokines) alongside a monocyte-skewed immune distribution. This pattern fits the broader literature describing a low-grade inflammatory phenotype and altered innate immune signatures in FES (26). Monocyte-related alterations are biologically plausible given evidence that monocyte subsets relate to brain structure and cognition, and that monocyte activation pathways (e.g., TLR4-related signaling) have been linked to cognitive deficits and white matter integrity in schizophrenia (27). In parallel, the KP profile biased toward relatively neurotoxic metabolism (higher KYN/TRP, higher QA/KYNA, and lower KYNA/3-HK) is consistent with the notion that inflammatory signaling induces IDO activity and diverts tryptophan metabolism toward kynurenines (28). Together, these baseline abnormalities are consistent with an “immune–KP axis” as a biologically coherent framework for early cognitive impairment.

Across follow-up, inflammatory markers and KP metabolites partially normalized and immune-cell distributions shifted toward healthy-control patterns. These trajectories are compatible with evidence that antipsychotic treatment can exert immunomodulatory effects, including reductions in pro-inflammatory cytokines in some studies and meta-analyses, although the magnitude and consistency of effects vary across markers and cohorts (29). Cross-sectional correlations further indicated that higher MCCB performance co-occurred with lower inflammatory burden and monocyte indices and with a relatively more protective KP balance, particularly KYNA/3-HK. This aligns with reports linking peripheral inflammatory signatures to MCCB-defined cognitive impairment in schizophrenia and with transdiagnostic evidence that innate immune dysregulation relates to cognitive heterogeneity (30). Mechanistically, inflammatory cytokines can influence glutamatergic signaling and synaptic plasticity, while kynurenines provide a direct biochemical bridge between immune activation and neuroactive metabolites (28). The convergence of inflammatory, monocyte, and KP correlates supports the concept that peripheral immune-metabolic state may index a “cognitive vulnerability” dimension in FES.

A key analytic contribution of this study is the decomposition of biomarkers into between-person (person-mean) and within-person (time-varying deviation) components. The predominance of significant between-person associations after FDR correction indicates that stable inter-individual differences in inflammatory burden, monocyte-related measures, and KP balance are more strongly linked to cognitive level than short-term within-person biomarker fluctuations. This pattern is consistent with emerging evidence that inflammatory–cognitive subgroups and immunophenotypes can show relative stability over time in severe mental illness (31). By contrast, change-score models suggested that cognitive improvement over follow-up was most reproducibly coupled to KP remodeling (Δ_KYNA_, Δ_KYNA/KYN_, Δ_KYNA/3-HK_, and Δ_3-HK_), in line with frameworks positioning KP as a mediator between immune activation and neurocognitive outcomes and with reviews emphasizing links between KP dysregulation and cognition in schizophrenia (32). The largest effect size observed for Δ_KYNA/3-HK_ at month 6 supports the view that this ratio may capture an important aspect of KP rebalancing during early treatment. This interpretation is biologically plausible because kynurenine-pathway metabolites have distinct neuroactive properties: KYNA exerts receptor-modulating effects, including antagonistic actions at NMDA-related signaling, whereas 3-HK is generally considered a pro-oxidative metabolite (33). In addition, central kynurenine-pathway alterations have been implicated in schizophrenia, supporting the relevance of KP dysregulation to disease biology (34). Therefore, an increase in KYNA/3-HK may reasonably be interpreted as reflecting a relative shift away from a more oxidative and biologically adverse metabolic profile toward a comparatively more protective balance. However, this interpretation remains inferential and should not be taken as direct evidence of enzymatic flux or a causal NMDA-mediated mechanism (35, 36). Rather, we interpret Δ_KYNA/3-HK_ as a composite marker consistent with KP rebalancing during early treatment, which may partly explain why it showed the strongest association with cognitive improvement in our cohort. Taken together, inflammation and monocyte measures may primarily stratify stable cognitive differences (between-person), whereas KP remodeling may better capture the immuno-metabolic component of interval cognitive improvement during early treatment. Additional exploratory mediation analyses further supported this framework by showing that the associations of several inflammatory markers with baseline cognition were statistically linked to variation in KYNA/3-HK, suggesting that KP imbalance may represent one pathway through which peripheral immune activation relates to cognitive dysfunction in FES, although the cross-sectional design precludes causal inference.

Several limitations warrant consideration. First, peripheral biomarkers may not directly mirror central immune or KP processes, and blood–brain correspondence may be imperfect. Second, healthy controls were assessed at baseline only, limiting inference regarding normative longitudinal variability. Third, treatment was protocolized (aripiprazole) only through week 6 and naturalistic thereafter. Although exploratory analyses using visit-specific CPZE and switch status did not show significant associations with KP change, cumulative antipsychotic exposure and medication-specific effects were not fully captured, and residual confounding by treatment changes, adherence, and lifestyle factors remains possible. Future studies should incorporate repeated control assessments, more frequent biomarker sampling, central proxies (e.g., imaging or CSF measures) when feasible, and immunophenotype stratification to test whether KP-targeted or anti-inflammatory adjuncts preferentially benefit cognitively impaired subgroups.

## Conclusion

In antipsychotic-naïve FES, cognitive impairment co-occurred with heightened inflammation, monocyte-related immune shifts, and KP dysregulation. Longitudinally, stable inter-individual differences in inflammatory/monocyte burden and KP balance tracked cognitive performance, whereas cognitive improvement was most consistently associated with KP remodeling. These findings highlight the immune–KP axis as a promising framework for future biomarker-guided stratification and mechanistically informed intervention research in FES.

## Data Availability

The original contributions presented in the study are included in the article/**Supplementary Material**. Further inquiries can be directed to the corresponding author.
