# Supplementary material for: Peripheral immune and kynurenine profiles are associated with cognitive change during early treatment of first-episode schizophrenia
Source: Front Psychiatry. 2026 Mar 25;17:1791018. doi: 10.3389/fpsyt.2026.1791018 (PMC13056681; doi:10.3389/fpsyt.2026.1791018)
Supplement: Supplementary file 1 [file Table1.docx]

**Supplementary Table 1. Exploratory analyses of antipsychotic exposure and kynurenine pathway metabolites**

| KP metabolite | Dose–response associations | | | | Sensitivity analysis, median Δ (IQR) | | |
| --- | --- | --- | --- | --- | --- | --- | --- |
|  | Week 6 (n = 136) | | Month 6 (n = 136) | |  |  |  |
|  | Spearman ρ | q (FDR) | Spearman ρ | q (FDR) | Aripiprazole (n=113) | Switched (n=23) | q (FDR) |
| TRP | 0.024 | 0.783 | 0.057 | 0.511 | 4.431 (-5.492, 15.496) | 6.670 (-7.877, 19.308) | 0.848 |
| KYN | 0.043 | 0.621 | 0.054 | 0.532 | -0.044 (-0.352, 0.441) | 0.093 (-0.338, 0.499) | 0.523 |
| KYN/TRP | 0.015 | 0.865 | -0.025 | 0.771 | -2.610 (-14.765, 6.111) | -3.737 (-8.844, 7.101) | 0.88 |
| KYNA | 0.153 | 0.075 | -0.107 | 0.216 | 10.575 (3.713, 18.291) | 10.455 (4.753, 17.753) | 0.844 |
| KYNA/KYN | 0.124 | 0.152 | -0.103 | 0.233 | 0.005 (-0.001, 0.008) | 0.006 (-0.002, 0.008) | 0.732 |
| 3-HK | -0.193 | 0.224 | 0.027 | 0.753 | -9.309 (-18.214, 3.247) | -6.421 (-9.668, 3.497) | 0.193 |
| QA | 0.111 | 0.198 | 0.045 | 0.604 | -73.881 (-164.538, 78.627) | -40.461 (-183.059, 68.253) | 0.889 |
| QA/KYNA | -0.046 | 0.596 | 0.068 | 0.432 | -3.033 (-5.763, -0.277) | -2.418 (-6.008, -0.893) | 0.912 |
| KYNA/3-HK | 0.155 | 0.338 | -0.123 | 0.155 | 0.275 (0.127, 0.418) | 0.208 (0.084, 0.321) | 0.204 |

Notes: CPZE, chlorpromazine equivalents; KP, kynurenine pathway; IQR, interquartile range. In the dose–response analysis, Spearman correlations were used to examine associations between time-matched antipsychotic exposure (CPZE, mg/day) and KP metabolite levels at week 6 and month 6. In the sensitivity analysis, changes in KP metabolites were calculated as month-6 minus baseline and compared between patients who remained on aripiprazole and those who switched to another antipsychotic by month 6. The switched group included quetiapine (*n* = 10), risperidone (*n* = 9), and clozapine (*n* = 4). Group comparisons were performed using the Mann–Whitney U test. Benjamini–Hochberg false discovery rate correction was applied separately within each time point across the 9 KP metabolites for the dose–response analysis, and across the 9 KP metabolites for the sensitivity analysis. KP, kynurenine pathway; TRP, tryptophan; KYN, kynurenine; KYNA, kynurenic acid; 3-HK, 3-hydroxykynurenine; QA, quinolinic acid.

**Supplementary Table 2. Spearman correlation coefficients between inflammatory markers and kynurenine pathway metabolites at baseline, week 6, and month 6**

| KP metabolite | IFN-γ | IL-10 | IL-8 | IL-1β | IL-6 | TNF-α | hs-CRP |
| --- | --- | --- | --- | --- | --- | --- | --- |
| **Baseline** |  |  |  |  |  |  |  |
| TRP | **-0.221** | 0.181 | **-0.228** | -0.061 | -0.130 | **-0.191** | -0.155 |
| KYN/TRP | **0.333** | **-0.266** | **0.241** | 0.064 | **0.265** | **0.272** | **0.308** |
| KYN | **0.247** | **-0.194** | 0.118 | 0.022 | **0.249** | 0.176 | **0.297** |
| KYNA | **-0.521** | **0.582** | **-0.398** | -0.112 | **-0.242** | **-0.190** | **-0.384** |
| KYNA/KYN | **-0.290** | **0.330** | **-0.241** | 0.022 | -0.183 | -0.113 | **-0.289** |
| 3-HK | **0.361** | **-0.413** | **0.290** | **0.321** | **0.259** | **0.209** | 0.165 |
| QA | **-0.200** | 0.145 | -0.054 | -0.160 | -0.052 | -0.065 | -0.036 |
| QA/KYNA | 0.109 | -0.182 | 0.163 | -0.082 | 0.089 | 0.036 | 0.177 |
| KYNA/3-HK | **-0.611** | **0.682** | **-0.476** | **-0.288** | **-0.320** | **-0.238** | **-0.378** |
| **Week 6** |  |  |  |  |  |  |  |
| TRP | 0.043 | 0.057 | -0.050 | 0.050 | -0.002 | 0.041 | -0.029 |
| KYN/TRP | -0.086 | -0.125 | 0.133 | -0.109 | 0.080 | -0.049 | 0.033 |
| KYN | -0.030 | -0.121 | 0.128 | -0.067 | 0.095 | 0.041 | 0.029 |
| KYNA | 0.051 | -0.135 | -0.034 | 0.117 | -0.054 | 0.194 | -0.039 |
| KYNA/KYN | -0.005 | -0.011 | 0.007 | 0.077 | 0.009 | 0.104 | 0.069 |
| 3-HK | -0.072 | -0.007 | -0.090 | -0.082 | 0.000 | -0.095 | 0.039 |
| QA | -0.030 | 0.048 | 0.054 | 0.006 | -0.009 | -0.004 | 0.077 |
| QA/KYNA | -0.056 | 0.102 | 0.048 | -0.071 | 0.008 | -0.114 | 0.081 |
| KYNA/3-HK | **-0.347** | 0.107 | **-0.409** | **-0.336** | **-0.314** | **-0.263** | -0.058 |
| **Month 6** |  |  |  |  |  |  |  |
| TRP | -0.073 | 0.062 | 0.017 | -0.258 | -0.060 | -0.156 | -0.047 |
| KYN/TRP | 0.122 | 0.003 | 0.032 | **0.344** | 0.203 | 0.209 | 0.104 |
| KYN | 0.059 | 0.122 | 0.000 | 0.189 | 0.172 | 0.082 | 0.110 |
| KYNA | -0.029 | -0.075 | -0.138 | -0.052 | 0.057 | 0.051 | -0.154 |
| KYNA/KYN | -0.129 | -0.061 | -0.056 | 0.001 | -0.075 | -0.150 | -0.137 |
| 3-HK | 0.013 | -0.101 | 0.014 | 0.038 | -0.009 | 0.058 | -0.077 |
| QA | -0.067 | -0.026 | 0.126 | 0.046 | 0.041 | -0.089 | 0.171 |
| QA/KYNA | -0.053 | 0.009 | 0.164 | 0.056 | 0.039 | -0.089 | 0.218 |
| KYNA/3-HK | -0.097 | 0.052 | -0.121 | -0.140 | -0.049 | 0.042 | 0.123 |

Notes: Values are Spearman’s rank correlation coefficients (rho). Bold values indicate correlations that remained statistically significant after Benjamini–Hochberg false discovery rate correction within each time point. KP, kynurenine pathway; TRP, tryptophan; KYN, kynurenine; KYNA, kynurenic acid; 3-HK, 3-hydroxykynurenine; QA, quinolinic acid
